# Genetic variations of *HvP5CS1* and their association with drought tolerance related traits in barley (*Hordeum vulgare* L.)

**DOI:** 10.1038/s41598-017-08393-0

**Published:** 2017-08-11

**Authors:** Yanshi Xia, Ronghua Li, Guihua Bai, Kadambot H. M. Siddique, Rajeev K. Varshney, Michael Baum, Guijun Yan, Peiguo Guo

**Affiliations:** 1 International Crop Research Center for Stress Resistance/College of Life Sciences, Guangzhou University, Guangzhou, 510006 China; 2 USDA-ARS Hard Winter Wheat Genetics Research Unit, 4008 Throckmorton Hall, Manhattan, KS 66506 USA; 3 The UWA Institute of Agriculture, The University of Western Australia, Perth, WA 6001 Australia; 4 International Crops Research Institute for the Semi-Arid Tropics (ICRISAT), Patancheru, 502324 Telangana State India; 5 International Center for Agricultural Research in the Dry Areas (ICARDA), PO.Box 114/5055, Beirut, Lebanon; 6 School of Agriculture and Environment, The University of Western Australia, Perth, WA 6001 Australia

## Abstract

Delta-1-pyrroline-5-carboxylate synthase gene1 (*P5CS1*) is the key gene involved in the biosynthesis of proline and is significantly induced by drought stress. The exploration of genetic variation in *HvP5CS1* may facilitate a better understanding of the mechanism of drought adaptation in barley. In the current study, 41 polymorphisms including 16 single nucleotide polymorphisms (SNPs) and 25 insertions/deletions (indels) were detected in *HvP5CS1* among 287 barley (*Hordeum vulgare* L.) accessions collected worldwide, with 13 distinct haplotypes identified in the barley collection. Five polymorphisms in *HvP5CS1* were significantly (*P* < 0.001) associated with drought tolerance related traits in barley. The phenotypic variation of a given trait explained by each associated polymorphism ranged from 4.43% to 9.81%. Two sequence variations that were significantly (*P* < 0.0001) associated with grain yield had marginally significant positive Tajima’s D values in the sliding window, so they might have been selected for environmental adaptation. Meanwhile, two haplotypes *HvP5CS1_*H1 and *HvP5CS1_*H4, which contained desired alleles of the two variations mentioned above, were significantly (*P* < 0.001) associated with drought tolerance related traits, and explained 5.00~11.89% of the phenotypic variations. These variations associated with drought tolerance related traits can be used as potential markers for improving drought tolerance in barley.

## Introduction

Drought is a major adverse climatic factor that severely restricts plant growth and sustainable agricultural development^1^. Breeding drought-tolerant cultivars has been a major objective of many crop improvement programs and is becoming an important strategy of adapting crops to climate changes^2, 3^.

Barley (*Hordeum vulgare* L.) is the fourth most grown cereal crop worldwide, and is characterized by having relatively high drought tolerance^4^. Consequently, barley is frequently used as a model crop for investigating the genetic basis of drought tolerance^5, 6^. Drought-adaptive traits derived from wild relatives and landraces of barley may contribute to increased yields and yield stability in improved cultivars under drought conditions^7^. The ability of different barley genotypes to adapt to drought stress resides in their genetic diversity^8, 9^. The exploration of genetic variation in genes related to drought response in natural populations may facilitate a better understanding of the molecular mechanisms of drought tolerance in barley.

Previous research identified the *P5CS* gene, encoding delta-1-pyrroline-5-carboxylate synthase (P5CS), as the main drought-responsive gene in barley^10–12^. In plants, P5CS is a rate-limiting enzyme involved in the biosynthesis of proline from glutamate, and proline is considered one of the most accumulated osmolytes for adaptation to drought stress^13, 14^. The P5CS protein is encoded by two genes, *P5CS1* and *P5CS2*, that have different expression patterns and perform distinct functions during crop development. Studies have shown that the expression of *P5CS1* is significantly induced by several abiotic stresses such as drought and salt^14–16^. However, *P5CS2* is apparently a housekeeping gene that mainly functions in basic proline metabolism in *Arabidopsis*, *Sorghum bicolor*, and *Zea mays* ^15–17^. Transgenic plants with overexpressed *P5CS1* significantly increased biomass under abiotic stress conditions compared with non-transgenic controls^18–20^. *Arabidopsis P5CS1* T-DNA insertion mutants reduced *P5CS1* transcription level, and proline synthesis, hypersensitivity to low water potential, and accumulation of reactive oxygen species^16^. Kesari *et al*.^21^ found that intron sequence variations in *Arabidopsis P5CS1* promoted alternative splicing, produced a non-functional *P5CS1* transcript variant, and demonstrated that *P5CS1* was a gene under selection for environmental adaptation. Barley genotypes with drought tolerance manifested higher proline contents and higher expression levels of *HvP5CS1* compared to drought-sensitive genotypes under drought stress^22, 23^. However, little is known about the underlying effect of genetic variations in *HvP5CS1* on traits related to drought tolerance.

Single nucleotide polymorphisms (SNPs) and small insertions and deletions (indels) are the most common forms of nucleotide variations in natural populations, and may be significantly associated with phenotypic variations and plant adaptation to diverse environments^24^. SNPs are ideal molecular markers for genome mapping, marker-assisted selection and association analyses in plant breeding programs^25–28^. Compared to the analysis of SNPs in arbitrary sequences, careful analysis of SNPs in genes of specific interest presents a much greater value for association analysis between allelic variants with phenotypic differences and the subsequent marker-assisted selection of the associated traits^29, 30^. To date, allelic variations of *HvP5CS1* in barley have not been systematically examined. Exploration of genetic variation in *HvP5CS1* may provide a better understanding of the functions of *HvP5CS1* and useful information to improve drought tolerance in barley.

In this study, EcoTILLING (a variant of Targeting Induced Local Lesions in Genomes) technology^31^ was used to detect allelic variation in the targeted region of *HvP5CS*1 in 287 barley accessions collected from diverse geographical areas, and to evaluate nucleotide diversity and the neutral test of detected regions. In addition, association analysis between the allelic variations and drought tolerance related traits was performed to validate gene function and provide a potential source of beneficial alleles for marker-assisted selection to improve barley growth under drought stress.

## Results

### Nucleotide polymorphisms in *HvP5CS1*

**Figure 1:**
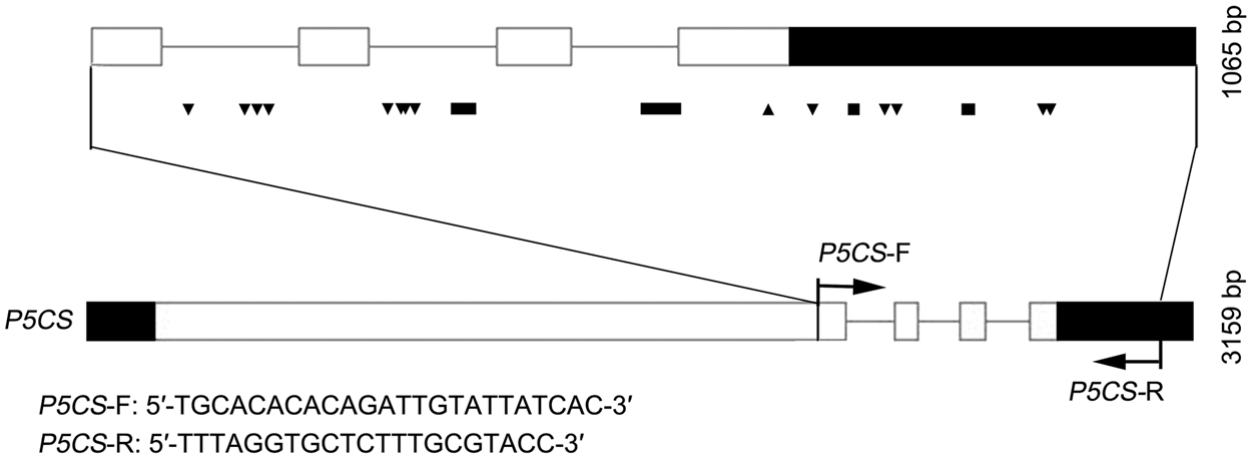
Diagram of PCR amplification and distribution of SNPs in *HvP5CS1*. White boxes are the coding sequence of exons, black boxes are the 5′-UTR and 3′-UTR, and black lines are introns. The relative positions of the PCR products amplified for EcoTILLING are indicated as arrows and black bars. Black up arrows indicate changes in coding regions of DNA and do not affect the amino acid sequence of the protein product. Black down arrows indicate changes in the non-coding regions of DNA. Black bars refer to either insertions or deletions.

Across the amplified region of *HvP5CS1*, 41 natural variation sites were identified among the 287 accessions by EcoTILLING analysis and sequencing. The polymorphism information content (PIC) values of those sites ranged from 0.014 to 0.483, with an average of 0.243 per site (Table 1). These variations contained 16 SNPs and 25 indels, with a mean of one SNP per 67 bp and one indel per 43 bp in the genomic sequence. Of the 16 SNPs, only one was located in the exon region of *HvP5CS1*, which was a silent synonymous change, while eight were in the intron region and seven were in the 3′ downstream of the non-coding region. Of the 25 indels, eight were in the exon regions, 11 in the intron regions and six in the 3′ downstream of the non-coding region (Table 1 and Fig. 1).

**Table 1:** Polymorphism information content (PIC) values and positions in the gene for nucleotide polymorphisms in *HvP5CS1*.

No.	Nucleotide change^a^	PIC	Position^b^	No.	Nucleotide change^a^	PIC	Position^b^
1	A98G	0.014	Intron	22	T570	0.342	Exon
2	G150T	0.434	Intron	23	GCA573	0.334	Exon
3	C162T	0.014	Intron	24	GC577	0.342	Exon
4	C174T	0.079	Intron	25	TGTCTC581	0.342	Exon
5	G288A	0.448	Intron	26	TGTCTC581:TGT	0.014	Exon
6	T302C	0.261	Intron	27	TTATA588	0.342	Exon
7	G305A	0.134	Intron	28	G656A	0.028	Exon
8	G315T	0.014	Intron	29	T697C	0.047	NC
9	CAA354	0.302	Intron	30	T735TT	0.483	NC
10	TATAT358	0.302	Intron	31	C736CT	0.483	NC
11	TTCT365	0.302	Intron	32	C737CC	0.483	NC
12	T537	0.437	Intron	33	G768C	0.398	NC
13	G539	0.437	Intron	34	T778A	0.014	NC
14	A541	0.437	Intron	35	G846AT	0.479	NC
15	ATT543	0.437	Intron	36	C847CG	0.479	NC
16	TG555	0.014	Intron	37	T848TT	0.479	NC
17	AT558	0.014	Intron	38	C847T	0.302	NC
18	T564	0.014	Intron	39	T917C	0.134	NC
19	T566	0.014	Intron	40	T920G	0.274	NC
20	TCT568	0.014	Exon	41	T926C	0.047	NC
21	CA578	0.014	Exon				

^a^The first letter indicates wild type nucleotide at mutation site from the reference DNA (ICARDA IG: 138264), followed by the position of the mutation based on PCR amplicon in reference DNA, and followed by mutated nucleotide from tested sample DNA. ^b^“NC” = “no-coding”.

Nucleotide diversity (π) of the targeted region of *HvP5CS1* was 0.00917 across 287 barley accessions. π values were different among geographic regions, ranging from 0.00683 for European accessions (nine accessions) to 0.00997 for Arabian Peninsula accessions (14 accessions) (Table 2). To test whether the identified nucleotide polymorphisms were selectively neutral, D*, F* and Tajima’s D statistic were calculated. For 287 barley accessions, Tajima’s D value was 1.45687, but not significant (*P* < 0.05). However, the values of D* and F* were highly significant (*P* < 0.02). Apart from the Australian and Europe accessions, the other subgroups, including those from Africa, Arabian Peninsula, Middle East Asia and North East Asia, showed evidence of non-neutral variation (Table 2). In addition, analysis of the *HvP5CS1* sequence in a sliding window plot for Tajima’s D value identified three regions with significant or marginally significant positive Tajima’s D values in the sliding windows between nucleotides 505–525, 705–745 and 825–865, respectively (Supplementary Fig. S1).

**Table 2:** Barley *HvP5CS1* nucleotide diversity (π), haplotype diversity and selection (D* and F*, and Tajima’s D) statistics for each geographic region.

Population^a^	Number of accessions	Number of polymorphic sites	Nucleotide diversity (π)	Number of haplotypes	Haplotpe diversity	D*	F*	Tajima’s D
Total	287	41	0.00917	13	0.823	2.22194**	2.27376**	1.45687
AFR	55	24	0.00829	6	0.742	1.43651	2.01297**	2.15811*
NEA	106	37	0.00785	11	0.740	1.78802**	1.50814	0.46904
MEA	55	28	0.00876	9	0.823	1.82735**	2.11911**	1.69491
APS	14	24	0.00997	5	0.824	1.38633*	1.68322*	1.68469
EUR	9	16	0.00683	3	0.667	0.37790	0.61811	1.09956
AUS	2	9	0.00851	2	—	—	—	—
UNK	46	28	0.00884	7	0.824	1.51791*	1.82388*	1.56938

^a^AFR: Africa, APS: Arabian Peninsula, AUS: Australia, EUR: Europe, MEA: Middle East Asia, NEA: North East Asia, UNK: country of origin not known. *P ≤ 0.05; **P ≤ 0.02.

The barley population was grouped into putative haplotype categories based on the cleaved banding pattern in evaluated gel-frames. For the 41 sequence-validated nucleotide variations across the amplified region of *HvP5CS1*, 13 distinct haplotypes were identified in 287 accessions (Supplementary Table S1), and eight of them (60%) were rare haplotypes with frequencies below 0.05. The *HvP5CS1_*H1 was the most-common haplotype with 78 accessions. The reference haplotype (*HvP5CS1_*H2) was the second most-common in amplicon, which included 61 accessions. The non-reference haplotype frequencies (NHF) ranged from 0.007 to 0.272, which differed significantly among the geographical regions of the tested accessions. Accessions from North East Asia had the highest NHFs while those from the Arabian Peninsula had the lowest (Supplementary Table S2).

### Population structure and genetic diversity

**Figure 2:**
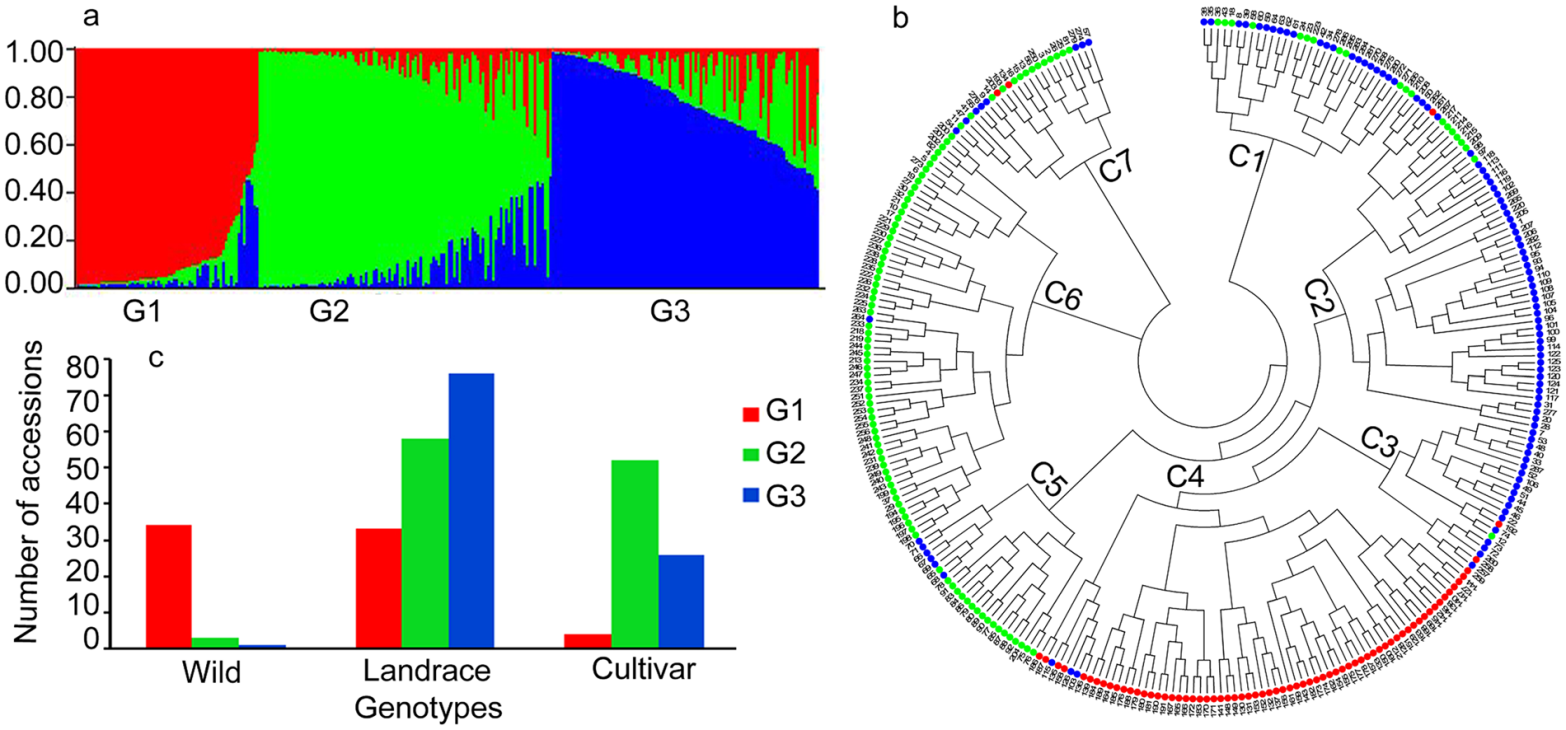
Genetic diversity of the 287 barley germplasm. (**a**) Population structure of the population. G1–G3 stand for three groups. The y-axis is the subgroup membership, and the x-axis is the accessions in three groups (G1, G2, and G3). (**b)** Cluster tree based on UPGMA. C1–C7 stand for cluster 1 to 7. The UPGMA tree is color-coded based on the results from population structure analysis (red G1, green G2, and blue G3); (**c**) Number of accessions in each of the three groups from different sources.

Association analysis requires the population structure to be taken into account in order to avoid false positive associations. Analysis of genetic distance and population structure confirmed a significant population structure in the barley population. The Bayesian approach implemented in the *Structure* software revealed a steadily increasing curve for LnP(D) (Supplementary Fig. S2), therefore, it is difficult to deduce the optimal number of subgroups based on the values of LnP(D). In contrast, the ΔK value attained a clear maximum at K = 3 (Supplementary Fig. S2) and the three groups (or clusters) revealed relatively low levels of admixture with G1, G2 and G3 containing 71, 113 and 103 accessions, respectively (Fig. 2a).

The results from the unweighted pair subgroup method with arithmetic (UPGMA) tree analysis on SSR marker data were generally consistent with the results from the STRUCTURE analysis with a few exceptions (Fig. 2b). UPGMA cluster C1, C2 and C3 contained 108 accessions with 85 from the G3, 20 from the G2 and three from the G1. The Cluster C4 contained 69 accessions with 66 from the G1 and three from the G3. Clusters C5, C6 and C7 contained 110 accessions with 93 from the G2, 15 from the G3 and two from the G1. Based on this information, we selected K = 3 as the optimal subgroup number. Of the ten runs for K = 3, the run with the highest likelihood value was selected to assign the posterior membership coefficients (Q-matrix) to each accession.

Among the 287 accessions tested, 34 (89.47%) wild accessions were classified into group G1, 52 cultivars (63.41%) into G2, 167 landraces into the three groups with 33 (19.76%) in G1, 58 (34.73%) in G2, and 76 (45.51%) in G3 (Fig. 2c).

### Association analysis between SNPs/haplotype and traits

Association analysis was performed to find possible associations between nucleotide variations in *HvP5CS1* and drought tolerance related agronomic traits. Since 28 of the nucleotide variations showed either linkage disequilibrium (LD) within groups or rare alleles (frequency <5%), only 13 distinct variations were used for association analysis. Of which, five were significantly (*P* < 0.001) associated with at least one of six phenotypic traits, and four were highly significant (*P* < 0.0001) (Table 3). The SNP at position 305 bp in the amplicon of *HvP5CS1* was significantly associated with days to heading (DH), total above ground biomass dry yield (BY) and grain yield (GY) at Tel Hadya location and plant height (PH) at Breda location, and explained 4.97%, 6.11%, 6.31% and 6.66% of the phenotypic variations, respectively. Another indel at position 735 bp exhibited significant associations with growth vigour (GV), GY and BY, and explained 6.83%, 6.65% and 6.62% of the phenotypic variations, respectively, at Tel Hadya, and 6.01% and 7.90% for GY and BY at Breda, respectively. Similarly, an indel at position 846 bp explained 6.98%, 7.71% and 7.71% of the phenotypic variations for GV, GY and BY at Tel Hadya, and 9.08% and 9.38% for GY and BY at Breda, respectively. The SNP at position 768 bp was significantly associated with spike length (SL), BY and GY, and explained 7.36%, 4.48% and 9.81% of the phenotypic variation at Tel Hadya, respectively, and 4.43% and 5.95% for SL and GY at Breda, respectively. The SNP at position 920 bp was significantly associated with DH, BY and GY at Tel Hadya and with PH at Breda, explaining 4.95%, 4.97%, 5.13% and 5.35% of the phenotypic variations, respectively. Therefore, the five SNPs significantly associated with drought tolerance related traits in this study may have potential as DNA markers to be used in marker-assisted selection for improving drought tolerance in barley after further validation.

**Table 3:** Significant associations between SNPs of *HvP5CS1* and agronomic traits of barley.

Site	Traits^a^	SNPs position^b^	F	P	R^2^ (%)	Desired allele	Frequency of desired allele
Tel Hadya (wet)	DH	305 G > A	11.37	8.78E-04	4.97*	A	15.86%
	BY	305 G > A	16.34	7.30E-05	6.11**	A	15.86%
	GY	305 G > A	17.8	3.58E-05	6.31**	A	15.86%
	GV	735 T < TT	18.35	2.73E-05	6.83**	TT	57.71%
	BY	735 T < TT	17.82	3.54E-05	6.62**	TT	57.71%
	GY	735 T < TT	18.83	2.17E-05	6.65**	TT	57.71%
	SL	768 G > C	18.73	2.28E-05	7.36**	G	77.53%
	BY	768 G > C	11.77	7.19E-04	4.48*	C	22.47%
	GY	768 G > C	28.96	2.00E-07	9.81**	C	22.47%
	GV	846 G < AT	18.79	2.21E-05	6.98**	AT	58.59%
	BY	846 G < AT	21.05	7.50E-06	7.71**	AT	58.59%
	GY	846 G < AT	22.13	4.50E-06	7.71**	AT	58.59%
	DH	920 T > G	11.82	6.99E-04	4.95*	G	16.74%
	BY	920 T > G	13.11	3.63E-04	4.97*	G	16.74%
	GY	920 T > G	14.27	2.04E-04	5.13*	G	16.74%
Breda (dry)	PH	305 G > A	17.54	4.05E-05	6.66**	G	84.14%
	BY	735 T < TT	14.47	1.84E-04	6.01*	TT	57.71%
	GY	735 T < TT	19.79	1.36E-05	7.90**	TT	57.71%
	GY	768 G > C	14.51	1.80E-04	5.95*	C	22.47%
	SL	768 G > C	11.19	9.66E-04	4.43*	G	77.53%
	BY	846 G < AT	23.44	2.40E-06	9.38**	AT	58.59%
	GY	846 G < AT	23.05	2.90E-06	9.08**	AT	58.59%
	PH	920 T > G	13.86	2.49E-04	5.35*	T	83.26%

^a^GV: growth vigour (1 = good; 5 = bad), DH: days to heading, PH: height at the base of the spike (cm), SL: spike length (cm), BY: total above ground biomass dry yield (kg/ha), GY: grain yield (kg/ha). ^b^The number of SNP positions is relative to the sequence on PCR amplicon of reference DNA (ICARDA IG: 138264). R^2^ is the fraction of the total variation explained by the marker. *Indicates SNP significantly (P < 0.001) associated with traits. **Indicates SNP highly significantly (P < 0.0001) associated with traits.

Haplotype-trait associations were restricted to haplotypes with a higher than 5% frequency of minor alleles. Two haplotypes were significantly (*P* < 0.001) associated with the phenotypic traits (Table 4), and contained desired alleles at four variation sites. The haplotype *HvP5CS1_*H1 showed highly significant (*P* < 0.0001) associations with GY and SL at Tel Hadya, and explained 11.89% and 7.55% of the phenotypic variations, respectively. Similarly, the haplotype *HvP5CS1_*H1 explained 5.23% and 5.30% of the phenotypic variations for GV and BY, respectively, at Tel Hadya, and 7.05% for GY at Breda. The haplotype *HvP5CS1_*H4 exhibited significant associations with BY at Tel Hadya and PH at Breda, and explained 5.00% and 5.92% of the phenotypic variations, respectively.

**Table 4:** Association results for significant (*P* < 0.001) haplotypes identified by the GLM for certain agronomic traits in 227 barley accessions.

Experiment site	Traits	Haplotype^a^	F	P	R^2^ (%)	Mean ± SD^b^
Tel Hadya (wet)	GY	*HvP5CS1_*H1 (51)	31.32	7.12E-08	11.89**	4720.2 ± 1111.99 (4311.3)
	SL	*HvP5CS1_*H1 (51)	17.12	5.15E-05	7.55**	7.3 ± 1.48 (8.2)
	BY	*HvP5CS1_*H1 (51)	12.36	5.43E-04	5.30*	10508.2 ± 1833.67 (10276.1)
	GV	*HvP5CS1_*H1 (51)	12.07	6.28E-04	5.23*	2.0 ± 0.69 (2.4)
	BY	*HvP5CS1_*H4 (36)	11.60	7.95E-04	5.00*	10417.8 ± 2727.46 (10311.3)
Breda (dry)	GY	*HvP5CS1_*H1 (51)	15.49	1.14E-04	7.05*	1558.6 ± 435.87 (1400.0)
	PH	*HvP5CS1_*H4 (36)	13.54	3.00E-04	5.92*	55.2 ± 7.29 (57.0)

^a^The numbers of accessions identified to carry the same haplotype are given in brackets. ^b^Weighted means for other haplotypes than targeted haplotypes (e.g. *HvP5CS1_*H1) are given in brackets.

## Discussion

Using transcriptome analysis, the *P5CS* gene has been cloned in various plant species^17, 32, 33^, but its allelic variation has only been characterized in common bean^13^. In this study, 41 natural variation sites were identified from 1065 bp DNA sequence of *HvP5CS1* across 287 barley accessions. The average frequency of polymorphisms was 67 bp per SNP and 43 bp per indel of the genomic sequence. The SNPs were distributed unevenly along the DNA sequence with nine variation sites in the exon regions, 19 in the intron regions and 13 in the 3′ downstream of the non-coding region. Several studies have shown that a low level of polymorphisms in exon regions but a high level in non-coding regions of barley^34–37^. Genetic diversity analysis in a new *P5CS* gene from common bean identified that the frequency of SNPs in intron sequences was 1.5-fold higher than in exon sequences. All indels were exclusively found in the intron region^13^. A C-terminal glutamic-γ-semialdehyde dehydrogenase (GSA-DH) domain in the *H*vP5CS1 protein, which is from 217–270 and 394– 444 nucleotide acids in the gene region, was an extremely conserved domain without any nucleotide variation, which agrees with Chen *et al*.^13^. Compared to previous reports on barley^38–40^, nucleotide diversity (π = 0.00917) and haplotype diversity (0.823) of *HvP5CS1* was relatively high in this study. As predicted, accessions from the Arabian Peninsula and Middle East Asia had higher nucleotide diversity (π) and haplotype diversity than those from the other five regions, which is consistent with previous reports^41, 42^. One plausible reason is that these geographic regions were the main distribution areas of wild barley^43^.

In this study, Tajima’s D value was not significant despites a high positive estimate of 1.45687, but the value of D* and F* was significant. These results may be attributed to the intermediate frequency of polymorphisms observed, which may result from balancing selection, diversifying selection or population subdivision^13^. In addition, analysis of the *HvP5CS1* sequence in a sliding window plot for Tajima’s D value illustrated that three regions had significantly or marginally significantly positive Tajima’s D values in the sliding windows between nucleotide midpoint numbers of 505–525, 705–745 and 825–865. The three domains may be the targets of naturally-acquired protective responses of barley under natural environments.

Association analysis emerged as a powerful approach to investigate the role of sequence polymorphisms in phenotype variation in response to environmental stresses^44^. In the present study, five variations in *HvP5CS1* were significantly (*P* < 0.001) associated with up to four drought tolerance related traits in barley. Of these, one at 305 bp of *HvP5CS1* was in an intron region, while the other four at positions 735 bp, 768 bp, 846 bp and 920 bp were at 3′ downstream of the non-coding region. These variations may affect the expression of *HvP5CS1*. In our previous studies, the transcription level of *HvP5CS1* in Tadmor (drought tolerant) was 1.22 fold that of WI2291 (drought sensitive) under drought stress^22^. Tadmor had positive alleles at the positions 305 bp, 735 bp and 846 bp in contrast with those in WI2291. Except for position 920, the other four sequence variations were significantly (*P* < 0.0001) associated with BY and GY at Tel Hadya (a favorable environment). Meanwhile two variations (at the positions 735 bp and 846 bp) were associated with BY and GY at Breda (a drought stress environment), which have marginally significantly positive Tajima’s D values in the sliding window. Haplotype-trait associations showed that two haplotypes, *HvP5CS1_*H1 and *HvP5CS1_*H4 carrying positive alleles of the two variations at positions 735 bp and 846 bp were significantly (*P* < 0.001) associated to drought tolerance related traits, and explained 5.00~11.89% of the phenotypic variations. Our previous research showed that Tadmor carrying *HvP5CS1_*H1 had high osmotic adjustments capacity and high yield stability, while Er/Apm and Express, both carrying *HvP5CS1_*H3, showed a low and constitutive osmotic capacity^45^. The variations at the positions 735 bp and 846 bp may provide useful markers as a selection tool to improve barley yield under stress conditions.

Several studies showed that GY was positively correlated with proline content in barley^46^ and wheat^47, 48^, and proline content had weak positive but non-significant correlations with DH, PH, SL and TKW in wheat^48^. Our study showed that GY had a significantly positive correlation with BY and TKW, but a significantly negative correlation with PH and SL in barley (Table 5), which agrees with several earlier reports^49–51^.

**Table 5:** Phenotypic scores from field trials for 227 barley accessions.

Site	Traits^a^	Range	Mean	SD^b^	Correlations					
					BY	GY	PH	SL	TKW	GV
Breda (dry)	BY	1270.0–6327.8	4235.4	997.88						
	GY	154.5–2625.4	1435.6	603.33	0.735**					
	PH	33.2–90.8	56.7	9.37	−0.216**	−0.388**				
	SL	4.4–12.6	8.2	1.61	−0.231**	−0.349**	0.394**			
	TKW	23.1–48.6	36.4	5.24	0.214**	0.338**	−0.079	−0.001		
Tel Hadya (wet)	BY	2686.1–14549.3	10328.2	2474.51						
	GY	153.2–7962.6	4403.1	1828.15	0.914**					
	PH	69.5–123.0	92.7	9.95	−0.130*	–0.223**				
	SL	3.4–13.6	8.0	1.89	−0.417**	–0.508**	0.137*			
	TKW	25.7–62.3	42.8	6.43	0.185**	0.193**	−0.002	0.139*		
	GV	0.9–5.2	2.3	1.02	−0.686**	−0.720**	0.269**	0.388**	−0.045	
	DH	72–104	85	5.26	0.202**	0.084	−0.025	−0.046	−0.244**	0.123

^a^BY: total above ground biomass dry yield (kg/ha), DH: days to heading, GV: growth vigour (1 = good, 5 = bad), GY: grain yield (kg/ha), PH: height at the base of the spike (cm), SL: spike length (cm), TKW: thousand kernel weight (g). ^b^SD: standard deviation. *Correlation is significant at the 0.05 level (2-tailed). **Correlation is significant at the 0.01 level (2-tailed).

This study identified 13 unique haplotypes based on 41 variations in *HvP5CS1* among 287 barley accessions collected from 35 countries using EcoTILLING technology. Five polymorphisms and two haplotypes were significantly (*P* < 0.001) associated with drought tolerance related traits in barley. These polymorphisms can serve as DNA markers in breeding to improve drought tolerance in barley after further validation.

## Materials and Methods

### Plant materials

A set of 287 barley (*Hordeum vulgare* L.) accessions consisting of 167 landraces, 82 cultivars and 38 wild accessions were obtained from the International Center for Agricultural Research in the Dry Areas (ICARDA). Among them, 241 were originally collected from 35 countries in six adjacent geographic regions, including Middle East Asia, North East Asia, Arabian Peninsula, Africa, Europe and Australia, while the origin of the other 46 accessions is unknown (Table 6 and Supplementary Table S3).

**Table 6:** The geographic origins of the barley accessions used in the study.

Continent	Geographical region	Countries
Africa (55)	Eastern (7)	Ethiopia (5), Eretria (2)
	Northern (48)	Morocco (5), Tunisia (6), Algeria (10), Egypt (14), Libya (13)
Asia (175)	Arabian Peninsula (14)	Oman (7), Saudi Arabia (4), Yemen (3)
	Middle East (55)	Iraq (4), Jordan (18), Lebanon (2), Palestine (2), Syria (29)
	North East (106)	Tajikistan (4), Turkmenistan (11), Uzbekistan (1), China (33), Pakistan (11), India (2), Azerbaijan (5), Afghanistan (9), Georgia(1), Cyprus (1), Turkey (7), Iran (21)
Europe (9)	Eastern (1)	Russia (1)
	Central (1)	Deutschland (1)
	Western (1)	France (1)
	Southern (6)	Greece (2), Albania (1), Bosnia and Herzegovina (2), Serbia and Montenegro (1)
Australia (2)		Australia (2)
Unknown (46)		Unkown countries of origin

Numbers in brackets indicate how many accessions from each country.

Genomic DNA was isolated from young leaf tissue of each accession using a modified cetyltrimethyl ammonium bromide (CTAB) protocol^52^, quantified using a spectrophotometer, and normalized to a concentration of 20 ng/μl.

### Evaluation of drought tolerance related traits

As described by Varshney *et al*.^53^, 227 barley accessions were grown in the 2004–2005 grown season at two locations in Syria—Tel Hadya (36°01′N; 37°20′E, elevation 300 m asl, long term average annual rainfall = 338 mm) and Breda (35°56′N; 37°10′E, elevation 354 m asl, long term average annual rainfall = 269 mm). Tel Hadya represented a favorable condition and Breda represented a drought stressed environment. Field plots were arranged in a row and column grid (10 rows, 28 columns). Trials were sown on 3 m^2^ plots with 1.2 m wide (4 rows 30 cm apart) and 2.5 m long under rainfed conditions. Rainfall at the growing season was 303 mm at Tel Hadya and 268 mm at Breda. Phenotypic data were recorded for seven developmental and yield-related traits: days to heading (DH in days), growth vigour (GV 1 = good to 5 = bad), plant height (PH in cm), spike length (SL in cm), 1000 kernel weight (TKW in g), grain yield (GY in kg/ha) and total above ground biomass dry yield (BY in kg/ha) as described in Varshney *et al*.^53^. These phenotypic traits were tested for correlations using SPSS 16.0 software (SPSS Inc., Chicago, IL, USA). Phenotypic scores of measured traits are listed in Table 5.

### PCR amplification and EcoTILLING assays

Natural variations in barley *HvP5CS1* were screened using EcoTILLING techniques. The primers were designed according to the mRNA sequence of *HvP5CS1* (GenBank:AK251855.1) with melting temperatures around 60 °C by using Primer 5.0 software (Premier Biosoft International, Palo Alto, CA, USA). The DNA region amplified by the primer pair is around 1065 bp, which includes a 3′-UTR and small coding sequence interrupted by three introns (Fig. 1). The forward primer labeled with IRDye700 at 5′-end and the reverse primer labeled with IRDye800 at 5′-end were synthesized by LI-COR Inc. (Lincoln, NE, USA).

The accession ICARDA IG 138264 (drought sensitive) was selected as a reference accession. PCR amplification of the target region was performed in a volume of 10 μl containing a mix of 10 ng each of reference and sample genomic DNAs. Heteroduplex formation, heteroduplex digestion, and denaturing polyacrylamide gel electrophoresis were performed as described by Xia *et al*.^54^. During electrophoresis, the LI-COR DNA Analyzer captured two images in IRD700 and IRD800 channels, respectively. Tiff images were manually scored using GelBuddy program^55^. Big dark bands of different sizes in both IRD700 and IRD800 channels were considered a polymorphic site (Supplementary Fig. S3). Total length PCR products from both channels should be equivalent to the fragment size of the undigested PCR product. Samples were grouped into putative haplotype categories based on the cleaved banding pattern in evaluated gel-frames.

### DNA sequencing and statistical analysis

Once a polymorphism in a barley accession was identified, the corresponding DNA sample was amplified with the primers used in EcoTILLING. The resulting PCR fragment was directly sequenced to confirm the polymorphisms. Each polymorphic site was sequenced from more than one accession that showed the polymorphism in the same site to confirm that only two alleles segregated at the specific site. Multiple sequence alignment was conducted using ClustalW software^56^. Nucleotide diversity (π), haplotype diversity, Tajima’s D^57^ and D* and F* values^58^ were calculated using DnaSP v5.0^59^. PIC was calculated as described in Kota *et al*.^60^.

### Population structure analysis

The barley germplasm collection was structured into geographical groups in which some individuals are possibly related, so background genetic effects must be controlled to avoid spurious associations. Incorporating structure components as covariates in association analysis helps to reduce the false associations^61^.

All of the accessions were genotyped using 21 SSR markers assigned to seven chromosomes in barley (Supplementary Table S4). Amplification of the SSRs was carried out as described by Hayden *et al*.^62^. Amplified PCR products were analyzed in the LI-COR 4300 DNA analyzer (LI-COR, Lincoln NE, USA) and scored by comparing sizes between PCR products and a molecular weight ladder. Data from the 21 SSR markers were used to determine the population structure using the STRUCTURE software version 2.3.3^63, 64^. The program was run for *K* ranging from 1 to 12 with ten replications per *K*, using the admixture model with 10,000 burn-in period and 100,000 iterations. The optimal number of subpopulations was determined by the *ad hoc* statistic *ΔK* based on the rate of change of the likelihood value^65^.

To validate the genetic structure and test for different models, genetic similarities for each pair of lines were calculated using the NTSYS-pc 2.11 software package (Biostatistics Inc., USA). Subsequently, a phylogenetic tree using the unweighted pair subgroup method with arithmetic means (UPGMA) based on the genetic similarities matrix was constructed using the MEGA 5.0 program^66^.

### Association analysis

Association between SNP markers in *HvP5CS1* and drought tolerance related traits was evaluated using a general linear model (GLM_*Q*) in the TASSEL v3.0 (http://www.maizegenetics.net/tassel), where the SNP was considered as a fixed effect, and the factor and matrix of subpopulation membership (Q matrix) were used as cofactors to account for population structure. The significance of associations between markers and traits was tested using an F-test. The association between a marker and a trait is represented by its R^2^ value, an estimate of the percentage of phenotypic variation explained by the marker.

As described above for single marker association analysis, haplotype-trait association test was also performed using GLM_*Q* in the TASSEL v3.0. Haplotypes with a frequency <5% were discarded to avoid biased associations. Only five haplotypes were identified for trait-haplotype association analysis. Accessions carrying the same haplotype were coded as “1”, whereas these accessions without the target haplotype were coded as “0” as described by Li *et al*.^67^. All other analyses were the same as described for single marker analysis.

### Data availability

All data generated or analysed during this study are included in this published article and its Supporting Information files.

## Electronic supplementary material

Supplementary Information
