# Supplementary material for: Genetic variations of HvP5CS1 and their association with drought tolerance related traits in barley (Hordeum vulgare L.)
Source: Sci Rep. 2017 Aug 11;7:7870. doi: 10.1038/s41598-017-08393-0 (PMC5554244; doi:10.1038/s41598-017-08393-0)
Supplement: Supplementary file 1 — Supplementary Information [file 41598_2017_8393_MOESM1_ESM.pdf]

Genetic variations of *HvP5CS1* and their association with drought tolerance related traits in barley (*Hordeum vulgare* L.)

Yanshi Xia[1,+], Ronghua Li[1,+], Guihua Bai[2], Kadambot H.M Siddique[3], Rajeev Varshney[4], Michael Baum[5], Guijun Yan[3,6] and Peiguo Guo[1]*

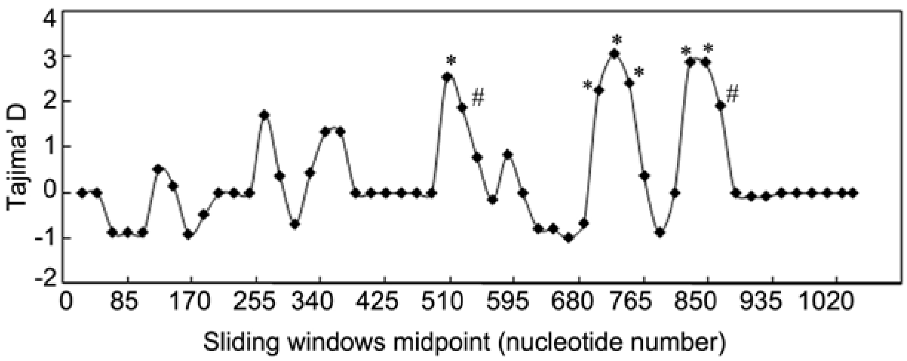

Figure S1. A sliding window plot of Tajima's D value in the targeted region of *HvP5CS1*. A window size of 50 nucleotides with a step size of 20 nucleotides was used to estimate Tajima's D using DnaSP 5.0. An asterisk (*) on the point of a measurement indicates significance at $P < 0.05$. The hash symbol (#) on the point of a measurement indicates significance at $P < 0.10$.

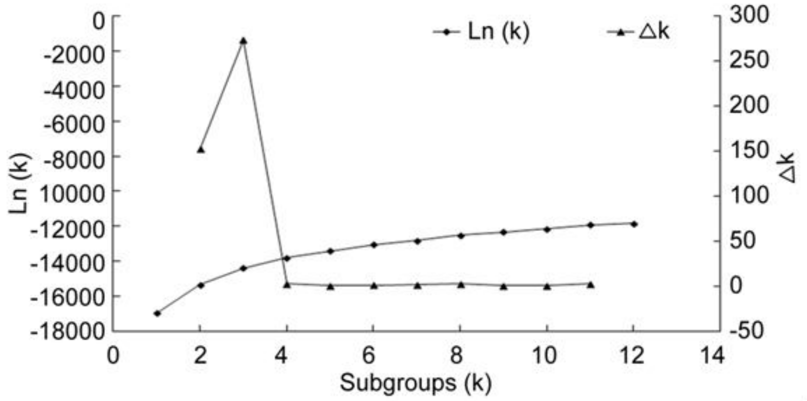

Figure S2. Comparison of statistical approaches used to determine the genetic background structure of the population. Ln(K) was calculated using Structure software, and *ΔK* was calculated as described by Evanno et al.[58].

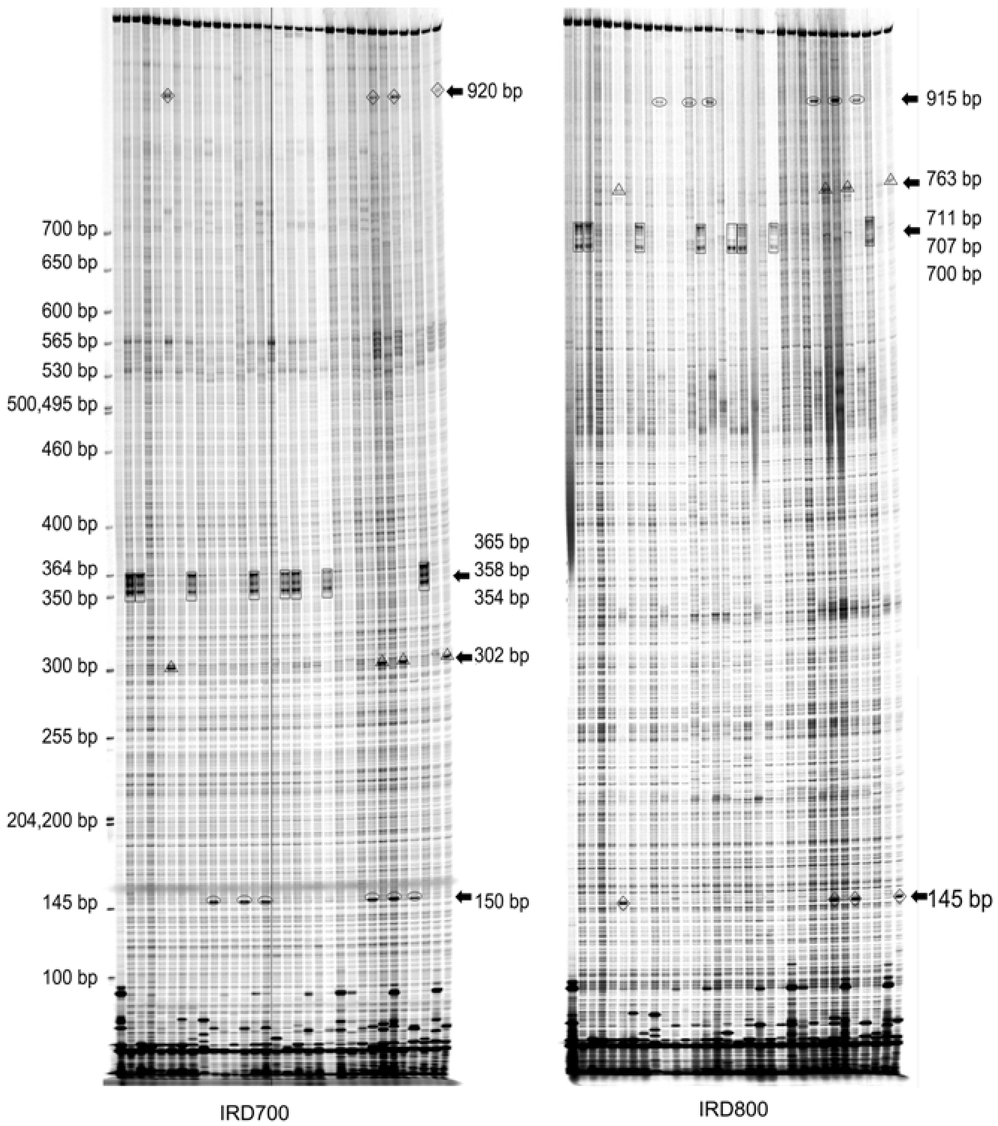

Figure S3. Detection of polymorphisms for part of the *HvP5CS1* gene sequences using EcoTILLING. The IR Dye 700 and IR Dye 800 channels are shown on the left and right, respectively. The specific cleavage products appear as intense bands. For any one nucleotide polymorphism, the sizes of the IRD 700 labeled and the IRD 800 labeled cleaved fragments should add up to the size of the uncut PCR product (1065 bp). Six nucleotide polymorphisms are shown, each with a corresponding fragment in the complementary fluorescent channel marked by the same box pattern. The sizes of the DNA ladders and digested PCR products are labeled with corresponding numbers and the units are bp.

Table S1 Distribution of polymorphic SNPs across 13 hylotypes. SNPs relative to the most common sequence are indicated in boldface. The number of SNP positions is relative to the sequence on PCR amplicon of reference DNA (ICARDA IG: 138264). A *horizontal dash* indicates the absence of the indicated bases.

Haplotypes	SNP position																			
	98	150	162	174	288	302	305	315	354	358	365	537	539	541	543	555	558	564	566	568
H1	A	**T**	C	C	**A**	T	G	G	CAA	TATAT	TTCT	–	–	–	–	TG	AT	T	T	TCT
H2	A	G	C	C	G	T	G	G	CAA	TATAT	TTCT	T	G	A	ATT	TG	AT	T	T	TCT
H3	A	G	C	C	G	T	G	G	–	–	–	T	G	A	ATT	TG	AT	T	T	TCT
H4	A	G	C	C	G	**C**	G	G	CAA	TATAT	TTCT	T	G	A	ATT	TG	AT	T	T	TCT
H5	A	G	C	C	G	T	**A**	G	CAA	TATAT	TTCT	T	G	A	ATT	TG	AT	T	T	TCT
H6	A	**T**	C	C	**A**	T	G	G	CAA	TATAT	TTCT	–	–	–	–	TG	AT	T	T	TCT
H7	A	G	C	**T**	G	T	G	G	CAA	TATAT	TTCT	T	G	A	ATT	TG	AT	T	T	TCT
H8	A	G	C	**T**	**A**	T	G	G	CAA	TATAT	TTCT	T	G	A	ATT	TG	AT	T	T	TCT
H9	A	G	C	C	G	T	G	G	–	–	–	T	G	A	ATT	TG	AT	T	T	TCT
H10	A	G	C	C	**A**	T	G	G	CAA	TATAT	TTCT	–	–	–	–	TG	AT	T	T	TCT
H11	**G**	G	C	C	G	T	G	**T**	CAA	TATAT	TTCT	T	G	A	ATT	TG	AT	T	T	TCT
H12	A	G	**T**	C	G	T	G	G	CAA	TATAT	TTCT	T	G	A	ATT	–	–	–	–	–
H13	A	**T**	C	C	G	T	**A**	G	CAA	TATAT	TTCT	T	G	A	ATT	TG	AT	T	T	TCT

Haplotypes	SNP position																			Total
	570	573	577	578	581	588	656	697	735	736	737	768	778	846	847	848	917	920	926	
H1	–	–	–	CA	–	–	G	T	TT	CT	CC	**C**	T	AT	CG	TT	T	T	T	78
H2	T	GCA	GC	CA	TGTCTC	TTATA	G	T	T	**C**	**C**	G	T	**G**	**C**	**T**	T	T	T	61
H3	–	–	–	CA	–	–	G	T	T	**C**	**C**	G	T	**G**	**T**	**T**	T	T	T	50
H4	–	–	–	CA	–	–	G	T	TT	CT	CC	G	T	AT	CG	TT	T	**G**	T	44
H5	–	–	–	CA	–	–	G	T	TT	CT	CC	G	T	AT	CG	TT	**C**	T	T	19
H6	–	–	–	CA	–	–	G	T	TT	CT	CC	G	T	AT	CG	TT	T	T	T	11
H7	–	–	–	CA	–	–	G	**C**	TT	CT	CC	G	T	AT	CG	TT	T	T	**C**	6
H8	–	–	–	CA	–	–	G	T	TT	CT	CC	G	T	AT	CG	TT	T	T	T	5
H9	–	–	–	CA	–	–	**A**	T	T	**C**	**C**	G	T	**G**	**T**	**T**	T	T	T	4
H10	–	–	–	CA	–	–	G	T	T	**C**	**C**	G	T	AT	CG	TT	T	**G**	T	3
H11	–	–	–	CA	–	–	G	T	TT	CT	CC	G	T	AT	CG	TT	T	T	T	2
H12	T	–	GC	–	TGT	TTATA	G	T	TT	CT	CC	G	**A**	AT	CG	TT	T	T	T	2
H13	–	–	–	CA	–	–	G	T	TT	CT	CC	G	T	AT	CG	TT	**C**	T	T	2

Table S2 Frequency of *HvP5CS1* haplotypes in different geographic barley growing regions.

Haplotype	Overall (287)	Geographic regions[a]						
		AFR (55)	APS (14)	AUS (2)	EUR (9)	MEA (55)	NEA (106)	UNK (46)
*HvP5CS1*_H1	0.272	0.031	**0.014**	–	–	0.031	**0.171**	0.024
*HvP5CS1*_H2	0.213	0.059	0.010	0.003	0.003	**0.063**	0.014	**0.059**
*HvP5CS1*_H3	0.174	0.024	**0.014**	0.003	**0.014**	0.014	0.059	0.045
*HvP5CS1*_H4	0.153	**0.070**	–	–	**0.014**	0.014	0.049	0.003
*HvP5CS1*_H5	0.066	–	–	–	–	0.035	0.014	0.017
*HvP5CS1*_H6	0.038	0.003	–	–	–	0.010	0.021	0.003
*HvP5CS1*_H7	0.021	–	–	–	–	–	0.014	0.007
*HvP5CS1*_H8	0.017	–	–	–	–	0.010	0.007	–
*HvP5CS1*_H9	0.014	0.003	0.001	–	–	–	0.003	–
*HvP5CS1*_H10	0.010	–	–	–	–	–	0.010	–
*HvP5CS1*_H11	0.007	–	–	–	–	0.007	–	–
*HvP5CS1*_H12	0.007	–	–	–	–	–	0.007	–
*HvP5CS1*_H13	0.007	–	–	–	–	0.007	–	–

[a]Haplotypes are ordered by overall frequency in all barley accessions. AFR: Africa, APS: Arabian Peninsula, AUS: Australia, EUR: Europe, MEA: Middle East Asia, NEA: North East Asia, UNK: country of origin not known. The number in brackets indicates the number of plants scored. Most frequent haplotypes within each population are highlighted in bold.

Table S3 General information on the barley accessions used in this study

ICARDA IG	Origin country[a]	Donor county	Genotype description (species)	Ear type
16981	TKM	USA	*Hordeum vulgare* subsp. vulgare convar. vulgare	6-row
17424	SCG	USA	*Hordeum vulgare* subsp. vulgare convar. vulgare	6-row
18767	TUR	USA	*Hordeum vulgare* subsp. vulgare convar. vulgare	6-row
18983	GRC	USA	*Hordeum vulgare* subsp. vulgare convar. vulgare	6-row
19390	CHN	USA	*Hordeum vulgare* subsp. vulgare convar. vulgare	6-row
19391	CHN	USA	*Hordeum vulgare* subsp. vulgare convar. vulgare	6-row
19453	IRN	USA	*Hordeum vulgare* subsp. vulgare convar. vulgare	6-row
19687	CHN	USA	*Hordeum vulgare* subsp. vulgare convar. vulgare	6-row
20905	AFG	USA	*Hordeum vulgare* subsp. vulgare convar. vulgare	6-row
23248	ETH	USA	*Hordeum vulgare* subsp. vulgare convar. vulgare	6-row
23515	ETH	USA	*Hordeum vulgare* subsp. vulgare convar. vulgare	6-row
24634	DZA	USA	*Hordeum vulgare* subsp. vulgare convar. vulgare	6-row
24745	AZE	USA	*Hordeum vulgare* subsp. vulgare convar. vulgare	6-row
24746	TKM	USA	*Hordeum vulgare* subsp. vulgare convar. vulgare	6-row
24751	CHN	USA	*Hordeum vulgare* subsp. vulgare convar. vulgare	6-row
24764	CHN	USA	*Hordeum vulgare* subsp. vulgare convar. vulgare	6-row
24774	CHN	USA	*Hordeum vulgare* subsp. vulgare convar. vulgare	6-row
24953	LBY	USA	*Hordeum vulgare* subsp. vulgare convar. vulgare	6-row
25704	IND	USA	*Hordeum vulgare* subsp. vulgare convar. vulgare	6-row
25839	EGY	USA	*Hordeum vulgare* subsp. vulgare convar. vulgare	6-row
25883	AFG	USA	*Hordeum vulgare* subsp. vulgare convar. vulgare	6-row
26002	AZE	USA	*Hordeum vulgare* subsp. vulgare convar. vulgare	6-row
26055	CHN	USA	*Hordeum vulgare* subsp. vulgare convar. vulgare	6-row
26056	CHN	USA	*Hordeum vulgare* subsp. vulgare convar. vulgare	6-row
26172	CHN	USA	*Hordeum vulgare* subsp. vulgare convar. vulgare	6-row
26178	CHN	USA	*Hordeum vulgare* subsp. vulgare convar. vulgare	6-row
26229	CHN	USA	*Hordeum vulgare* subsp. vulgare convar. vulgare	6-row
26276	IRQ	USA	*Hordeum vulgare* subsp. vulgare convar. vulgare	6-row
26727	AZE	USA	*Hordeum vulgare* subsp. vulgare convar. vulgare	6-row
27076	CHN	USA	*Hordeum vulgare* subsp. vulgare convar. vulgare	6-row
27630	PAK	USA	*Hordeum vulgare* subsp. vulgare convar. vulgare	6-row
27649	IRN	USA	*Hordeum vulgare* subsp. vulgare convar. vulgare	6-row
27653	IRN	USA	*Hordeum vulgare* subsp. vulgare convar. vulgare	6-row
27683	AFG	USA	*Hordeum vulgare* subsp. vulgare convar. vulgare	6-row
27773	IRN	USA	*Hordeum vulgare* subsp. vulgare convar. vulgare	6-row
27790	IRN	USA	*Hordeum vulgare* subsp. vulgare convar. vulgare	6-row
27794	IRN	USA	*Hordeum vulgare* subsp. vulgare convar. vulgare	6-row
27799	AFG	USA	*Hordeum vulgare* subsp. vulgare convar. vulgare	6-row
27803	AFG	USA	*Hordeum vulgare* subsp. vulgare convar. vulgare	6-row
29097	JOR	SYR	*Hordeum vulgare* subsp. vulgare convar. vulgare	6-row
31406	SYR	SYR	*Hordeum vulgare* subsp. vulgare convar. vulgare	6-row

31410	SYR	SYR	*Hordeum vulgare* subsp. vulgare convar. vulgare	6-row
31870	MAR	MAR	*Hordeum vulgare* subsp. vulgare convar. vulgare	6-row
31876	MAR	MAR	*Hordeum vulgare* subsp. vulgare convar. vulgare	6-row
31923	MAR	MAR	*Hordeum vulgare* subsp. vulgare convar. vulgare	6-row
31938	MAR	MAR	*Hordeum vulgare* subsp. vulgare convar. vulgare	6-row
32475	EGY	SYR	*Hordeum vulgare* subsp. vulgare convar. vulgare	6-row
32482	EGY	SYR	*Hordeum vulgare* subsp. vulgare convar. vulgare	6-row
32488	EGY	SYR	*Hordeum vulgare* subsp. vulgare convar. vulgare	6-row
32601	PAK	SYR	*Hordeum vulgare* subsp. vulgare convar. vulgare	6-row
32608	PAK	SYR	*Hordeum vulgare* subsp. vulgare convar. vulgare	6-row
32618	PAK	SYR	*Hordeum vulgare* subsp. vulgare convar. vulgare	6-row
32708	SYR	SYR	*Hordeum vulgare* subsp. vulgare convar. vulgare	6-row
32711	SYR	SYR	*Hordeum vulgare* subsp. vulgare convar. vulgare	6-row
32826	OMN	ITA	*Hordeum vulgare* subsp. vulgare convar. vulgare	6-row
32954	OMN	GBR	*Hordeum vulgare* subsp. vulgare convar. vulgare	6-row
32962	OMN	ITA	*Hordeum vulgare* subsp. vulgare convar. vulgare	6-row
32971	OMN	ITA	*Hordeum vulgare* subsp. vulgare convar. vulgare	6-row
32977	OMN	ITA	*Hordeum vulgare* subsp. vulgare convar. vulgare	6-row
33024	DZA	SYR	*Hordeum vulgare* subsp. vulgare convar. vulgare	6-row
33055	DZA	SYR	*Hordeum vulgare* subsp. vulgare convar. vulgare	6-row
33102	DZA	SYR	*Hordeum vulgare* subsp. vulgare convar. vulgare	6-row
33195	CHN	CHN	*Hordeum vulgare* subsp. vulgare convar. vulgare	6-row
33608	CHN	CHN	*Hordeum vulgare* subsp. vulgare convar. vulgare	6-row
33649	CHN	CHN	*Hordeum vulgare* subsp. vulgare convar. vulgare	6-row
34263	CHN	CHN	*Hordeum vulgare* subsp. vulgare convar. vulgare	6-row
35382	DZA	SYR	*Hordeum vulgare* subsp. vulgare convar. vulgare	6-row
35385	DZA	SYR	*Hordeum vulgare* subsp. vulgare convar. vulgare	6-row
35386	DZA	SYR	*Hordeum vulgare* subsp. vulgare convar. vulgare	6-row
35792	CHN	CAN	*Hordeum vulgare* subsp. vulgare convar. vulgare	6-row
35794	CHN	CAN	*Hordeum vulgare* subsp. vulgare convar. vulgare	6-row
35800	CHN	CAN	*Hordeum vulgare* subsp. vulgare convar. vulgare	6-row
35803	CHN	CAN	*Hordeum vulgare* subsp. vulgare convar. vulgare	6-row
35806	CHN	CAN	*Hordeum vulgare* subsp. vulgare convar. vulgare	6-row
35808	CHN	CAN	*Hordeum vulgare* subsp. vulgare convar. vulgare	6-row
35814	CHN	CAN	*Hordeum vulgare* subsp. vulgare convar. vulgare	6-row
35820	CHN	CAN	*Hordeum vulgare* subsp. vulgare convar. vulgare	6-row
35822	CHN	CAN	*Hordeum vulgare* subsp. vulgare convar. vulgare	6-row
35823	CHN	CAN	*Hordeum vulgare* subsp. vulgare convar. vulgare	2-row
35826	CHN	CAN	*Hordeum vulgare* subsp. vulgare convar. vulgare	6-row
36052	LBY	SYR	*Hordeum vulgare* subsp. vulgare convar. vulgare	6-row
37525	PAK	USA	*Hordeum vulgare* subsp. vulgare convar. vulgare	6-row
37554	LBY	ITA	*Hordeum vulgare* subsp. vulgare convar. vulgare	6-row
37556	LBY	ITA	*Hordeum vulgare* subsp. vulgare convar. vulgare	6-row
37576	LBY	ITA	*Hordeum vulgare* subsp. vulgare convar. vulgare	6-row

37726	TUN	TUN	*Hordeum vulgare* subsp. vulgare convar. vulgare	6-row
37729	TUN	TUN	*Hordeum vulgare* subsp. vulgare convar. vulgare	6-row
37784	TUN	TUN	*Hordeum vulgare* subsp. vulgare convar. vulgare	6-row
37813	TUN	TUN	*Hordeum vulgare* subsp. vulgare convar. vulgare	6-row
38214	CHN	CAN	*Hordeum vulgare* subsp. vulgare convar. vulgare	6-row
107010	IRN	SYR	*Hordeum vulgare* subsp. vulgare convar. vulgare	6-row
107020	IRN	SYR	*Hordeum vulgare* subsp. vulgare convar. vulgare	6-row
108499	PAK	SYR	*Hordeum vulgare* subsp. vulgare convar. vulgare	6-row
108911	IRQ	IRQ	*Hordeum vulgare* subsp. vulgare convar. vulgare	6-row
112483	GEO	DEU	*Hordeum vulgare* subsp. vulgare convar. vulgare	6-row
112715	IRN	DEU	*Hordeum vulgare* subsp. vulgare convar. vulgare	6-row
112840	LBY	DEU	*Hordeum vulgare* subsp. vulgare convar. vulgare	6-row
112865	LBY	DEU	*Hordeum vulgare* subsp. vulgare convar. vulgare	6-row
112931	TUR	DEU	*Hordeum vulgare* subsp. vulgare convar. vulgare	6-row
113084	SAU	ITA	*Hordeum vulgare* subsp. vulgare convar. vulgare	2-row
113120	IRN	IRN	*Hordeum vulgare* subsp. vulgare convar. vulgare	6-row
113126	IRN	IRN	*Hordeum vulgare* subsp. vulgare convar. vulgare	6-row
113128	IRN	IRN	*Hordeum vulgare* subsp. vulgare convar. vulgare	6-row
115919	LBY	DEU	*Hordeum vulgare* subsp. vulgare convar. vulgare	6-row
120565	TKM	RUS	*Hordeum vulgare* subsp. vulgare convar. vulgare	6-row
123901	UZB	SYR	*Hordeum vulgare* subsp. vulgare convar. vulgare	6-row
125827	AZE	RUS	*Hordeum vulgare* subsp. vulgare convar. vulgare	6-row
128122	IRN	SYR	*Hordeum vulgare* subsp. vulgare convar. vulgare	6-row
128124	IRN	SYR	*Hordeum vulgare* subsp. vulgare convar. vulgare	6-row
128125	IRN	SYR	*Hordeum vulgare* subsp. vulgare convar. vulgare	6-row
128133	IRN	SYR	*Hordeum vulgare* subsp. vulgare convar. vulgare	6-row
128158	PAK	SYR	*Hordeum vulgare* subsp. vulgare convar. vulgare	6-row
128159	PAK	SYR	*Hordeum vulgare* subsp. vulgare convar. vulgare	6-row
128160	PAK	SYR	*Hordeum vulgare* subsp. vulgare convar. vulgare	6-row
128170	DZA	SYR	*Hordeum vulgare* subsp. vulgare convar. vulgare	6-row
128187	EGY	SYR	*Hordeum vulgare* subsp. vulgare convar. vulgare	6-row
128199	JOR	SYR	*Hordeum vulgare* subsp. vulgare convar. vulgare	6-row
128204	EGY	SYR	*Hordeum vulgare* subsp. vulgare convar. vulgare	6-row
128218	LBY	SYR	*Hordeum vulgare* subsp. vulgare convar. vulgare	6-row
135258	JOR	SYR	*Hordeum vulgare* subsp. vulgare convar. vulgare	6-row
135528	TKM	SYR	*Hordeum vulgare* subsp. vulgare convar. vulgare	6-row
137761	TJK	SYR	*Hordeum vulgare* subsp. vulgare convar. vulgare	6-row
22957	ETH	USA	*Hordeum vulgare* subsp. vulgare convar. distichon	2-row
27892	SAU	USA	*Hordeum vulgare* subsp. vulgare convar. distichon	2-row
17406	BIH	USA	*Hordeum vulgare* subsp. vulgare convar. vulgare	2-row
17410	BIH	USA	*Hordeum vulgare* subsp. vulgare convar. vulgare	2-row
19620	ALB	USA	*Hordeum vulgare* subsp. vulgare convar. vulgare	2-row
20900	AFG	USA	*Hordeum vulgare* subsp. vulgare convar. vulgare	2-row
22912	ETH	USA	*Hordeum vulgare* subsp. vulgare convar. vulgare	2-row

24720	TKM	USA	*Hordeum vulgare* subsp. vulgare convar. vulgare	2-row
25095	CHN	USA	*Hordeum vulgare* subsp. vulgare convar. vulgare	2-row
25327	CHN	USA	*Hordeum vulgare* subsp. vulgare convar. vulgare	2-row
25710	IND	USA	*Hordeum vulgare* subsp. vulgare convar. vulgare	2-row
25843	EGY	USA	*Hordeum vulgare* subsp. vulgare convar. vulgare	2-row
25947	CHN	USA	*Hordeum vulgare* subsp. vulgare convar. vulgare	2-row
25961	CHN	USA	*Hordeum vulgare* subsp. vulgare convar. vulgare	2-row
27655	IRN	USA	*Hordeum vulgare* subsp. vulgare convar. vulgare	2-row
27784	IRN	USA	*Hordeum vulgare* subsp. vulgare convar. vulgare	2-row
28674	TUR	USA	*Hordeum vulgare* subsp. vulgare convar. vulgare	2-row
28693	TUR	USA	*Hordeum vulgare* subsp. vulgare convar. vulgare	2-row
28865	DEU	USA	*Hordeum vulgare* subsp. vulgare convar. vulgare	2-row
29057	SYR	SYR	*Hordeum vulgare* subsp. vulgare convar. vulgare	2-row
31396	SYR	SYR	*Hordeum vulgare* subsp. vulgare convar. vulgare	2-row
31412	SYR	SYR	*Hordeum vulgare* subsp. vulgare convar. vulgare	2-row
32687	EGY	SYR	*Hordeum vulgare* subsp. vulgare convar. vulgare	2-row
32694	EGY	SYR	*Hordeum vulgare* subsp. vulgare convar. vulgare	2-row
32756	SYR	SYR	*Hordeum vulgare* subsp. vulgare convar. vulgare	2-row
32774	SYR	SYR	*Hordeum vulgare* subsp. vulgare convar. vulgare	2-row
32812	EGY	SYR	*Hordeum vulgare* subsp. vulgare convar. vulgare	2-row
32814	OMN	ITA	*Hordeum vulgare* subsp. vulgare convar. vulgare	2-row
32978	OMN	ITA	*Hordeum vulgare* subsp. vulgare convar. vulgare	2-row
33094	SYR	SYR	*Hordeum vulgare* subsp. vulgare convar. vulgare	2-row
35220	SYR	SYR	*Hordeum vulgare* subsp. vulgare convar. vulgare	2-row
35236	SYR	SYR	*Hordeum vulgare* subsp. vulgare convar. vulgare	2-row
36058	TJK	SYR	*Hordeum vulgare* subsp. vulgare convar. vulgare	2-row
37608	YEM	ITA	*Hordeum vulgare* subsp. vulgare convar. vulgare	2-row
37612	YEM	ITA	*Hordeum vulgare* subsp. vulgare convar. vulgare	2-row
112781	IRN	DEU	*Hordeum vulgare* subsp. vulgare convar. vulgare	2-row
113076	SAU	ITA	*Hordeum vulgare* subsp. vulgare convar. vulgare	2-row
113082	SAU	ITA	*Hordeum vulgare* subsp. vulgare convar. vulgare	2-row
113095	YEM	ITA	*Hordeum vulgare* subsp. vulgare convar. vulgare	2-row
128088	AFG	SYR	*Hordeum vulgare* subsp. vulgare convar. vulgare	2-row
128172	SYR	SYR	*Hordeum vulgare* subsp. vulgare convar. vulgare	2-row
128173	SYR	SYR	*Hordeum vulgare* subsp. vulgare convar. vulgare	2-row
128200	JOR	SYR	*Hordeum vulgare* subsp. vulgare convar. vulgare	2-row
128202	JOR	SYR	*Hordeum vulgare* subsp. vulgare convar. vulgare	2-row
131668	TJK	SYR	*Hordeum vulgare* subsp. vulgare convar. vulgare	2-row
38215	CHN	CAN	*Hordeum vulgare* subsp. spontaneum	2-row
38611	SYR	SYR	*Hordeum vulgare* subsp. spontaneum	2-row
38638	SYR	ITA	*Hordeum vulgare* subsp. spontaneum	2-row
38660	AFG	USA	*Hordeum vulgare* subsp. spontaneum	2-row
38669	AFG	USA	*Hordeum vulgare* subsp. spontaneum	2-row
38672	TUR	USA	*Hordeum vulgare* subsp. spontaneum	2-row

38693	PAK	SYR	*Hordeum vulgare* subsp. spontaneum	2-row
38956	PAL	USA	*Hordeum vulgare* subsp. spontaneum	2-row
39126	PAL	USA	*Hordeum vulgare* subsp. spontaneum	2-row
39540	LBN	USA	*Hordeum vulgare* subsp. spontaneum	2-row
39802	PAK	SWE	*Hordeum vulgare* subsp. spontaneum	2-row
39847	SYR	SYR	*Hordeum vulgare* subsp. spontaneum	2-row
39857	SYR	SYR	*Hordeum vulgare* subsp. spontaneum	2-row
39891	EGY	SYR	*Hordeum vulgare* subsp. spontaneum	2-row
40021	JOR	SYR	*Hordeum vulgare* subsp. spontaneum	2-row
40022	JOR	SYR	*Hordeum vulgare* subsp. spontaneum	2-row
40031	JOR	SYR	*Hordeum vulgare* subsp. spontaneum	2-row
40034	JOR	SYR	*Hordeum vulgare* subsp. spontaneum	2-row
40035	JOR	SYR	*Hordeum vulgare* subsp. spontaneum	2-row
40039	JOR	SYR	*Hordeum vulgare* subsp. spontaneum	2-row
40056	JOR	SYR	*Hordeum vulgare* subsp. spontaneum	2-row
40059	JOR	SYR	*Hordeum vulgare* subsp. spontaneum	2-row
40064	JOR	SYR	*Hordeum vulgare* subsp. spontaneum	2-row
40071	JOR	SYR	*Hordeum vulgare* subsp. spontaneum	2-row
40072	JOR	SYR	*Hordeum vulgare* subsp. spontaneum	2-row
40082	SYR	SYR	*Hordeum vulgare* subsp. spontaneum	2-row
40101	TKM	SYR	*Hordeum vulgare* subsp. spontaneum	2-row
40104	TKM	SYR	*Hordeum vulgare* subsp. spontaneum	2-row
107046	IRN	SYR	*Hordeum vulgare* subsp. spontaneum	2-row
107427	IRQ	SYR	*Hordeum vulgare* subsp. spontaneum	2-row
110742	SYR	SYR	*Hordeum vulgare* subsp. spontaneum	2-row
112787	IRN	DEU	*Hordeum vulgare* subsp. spontaneum	2-row
115781	JOR	SYR	*Hordeum vulgare* subsp. spontaneum	2-row
120794	TKM	JPN	*Hordeum vulgare* subsp. spontaneum	2-row
132606	AZE	SYR	*Hordeum vulgare* subsp. spontaneum	2-row
135507	TKM	SYR	*Hordeum vulgare* subsp. spontaneum	2-row
135536	TKM	SYR	*Hordeum vulgare* subsp. spontaneum	2-row
135624	TKM	SYR	*Hordeum vulgare* subsp. spontaneum	2-row
138223	UNK	SYR	Zanbakian	2-row
138218	UNK	SYR	WI 2291	2-row
138272	UNK	SYR	UM (Zanbaka//SLB45-40/H.spont.41-1)	2-row
138274	UNK	SYR	UM (SLB39-39/H.spont.41-5)	2-row
138273	UNK	SYR	UM (SLB12-59//SLB45-40/H.spont.41-5)	2-row
138277	UNK	SYR	UM (SLB05-96/H.spont.41-5)	2-row
138276	UNK	SYR	UM (SLB05-96//H.spont.41-1/Tadmor)	2-row
138270	UNK	SYR	UM (PI386540/ArabiAbiad//H.spont.41-1/Tadmor)	2-row
138255	LBY	SYR	UM (M126/CM67//As/Pro/3/Alanda)	6-row
138271	UNK	SYR	UM (Hml//H.spont.41-1/Tadmor)	2-row
138214	UNK	SYR	UM (Harmal-02//Esp/1808-4L (P2))	2-row
138275	UNK	SYR	UM (H.spont.41-5/Tadmor//Hml-02/Lignee131)	2-row

138268	UNK	SYR	UM (H.spont.41-1/Tadmor)	2-row
138266	UNK	SYR	UM (Arta//H.spont.41-5/Tadmor)	2-row
138267	UNK	SYR	UM (Arta//H.spont.41-5/Tadmor)	2-row
138211	UNK	SYR	UM	2-row
138212	UNK	SYR	UM	2-row
138213	UNK	SYR	UM	2-row
138269	UNK	SYR	UM	2-row
138220	UNK	SYR	SLB 05-96	2-row
138242	UNK	SYR	Salmas	2-row
138251	UNK	SYR	Sadik-2	2-row
138250	UNK	SYR	Sadik-1	2-row
138252	UNK	SYR	Pamir 9	2-row
138221	UNK	SYR	Moroc 9-75	2-row
138240	UNK	SYR	Matnan-01	6-row
138224	UNK	SYR	Harmal	2-row
138217	UNK	SYR	ER/Apm	2-row
138237	TUR	SYR	CV (Tokak)	2-row
138230	DZA	SYR	CV (Tichedrett)	6-row
138265	LBY	SYR	CV (Tarida)	2-row
138262	ETH	SYR	CV (Shege)	6-row
138229	DZA	SYR	CV (Saida)	6-row
138236	JOR	SYR	CV (Rum)	6-row
138238	RUS	SYR	CV (Radical)	2-row
138228	TUN	SYR	CV (Martin)	6-row
138244	CYP	SYR	CV (Mari/Aths*2)	6-row
138231	TUN	SYR	CV (Manel)	6-row
138257	LBN	SYR	CV (Litani)	2-row
138260	AUS	SYR	CV (Keel)	2-row
138253	LBY	SYR	CV (Katara)	6-row
138234	IRQ	SYR	CV (IPA7)	6-row
138247	EGY	SYR	CV (Giza 126)	6-row
138246	EGY	SYR	CV (Giza 125)	6-row
138259	SYR	SYR	CV (Furat3)	2-row
138249	SYR	SYR	CV (Furat 2)	2-row
138248	SYR	SYR	CV (Furat 1)	6-row
138233	FRA	SYR	CV (Express)	2-row
138263	ERI	SYR	CV (Demhay)	2-row
138243	EGY	SYR	CV (CalM)	6-row
138239	TUR	SYR	CV (Bulbul)	2-row
138261	AUS	SYR	CV (Barque)	2-row
138254	LBY	SYR	CV (Barjouj)	6-row
138264	ERI	SYR	CV (Atsa)	2-row
138245	GRC	SYR	CV (Aths)	6-row
138235	MAR	SYR	CV (Arig8)	6-row

138215	SYR	SYR	CV	2-row
138216	SYR	SYR	CV	2-row
138219	SYR	SYR	CV	2-row
138222	SYR	SYR	CV	2-row
138225	SYR	SYR	CV	2-row
138226	SYR	SYR	CV	6-row
140405	TJK	SYR	CV	2-row
BMZ05-228	UNK	SYR	CV	2-row
BMZ05-229	UNK	SYR	CV	2-row
BMZ05-230	UNK	SYR	CV	6-row
BMZ05-231	UNK	SYR	CV	2-row
BMZ05-232	UNK	SYR	CV	2-row
BMZ05-233	UNK	SYR	CV	2-row
BMZ05-234	UNK	SYR	CV	2-row
BMZ05-235	UNK	SYR	CV	6-row
BMZ05-236	UNK	SYR	CV	2-row
BMZ05-237	UNK	SYR	CV	6-row
BMZ05-238	UNK	SYR	CV	6-row
BMZ05-239	UNK	SYR	CV	6-row
BMZ05-240	UNK	SYR	CV	2-row
BMZ05-241	UNK	SYR	CV	6-row
138258	UNK	SYR	Birlik	2-row
138256	UNK	SYR	Batal-1	2-row
138232	UNK	SYR	Badia	6-row
138241	UNK	SYR	Assala-04	6-row
138227	UNK	SYR	Alanda-01	6-row

[a]Standarad code for country of origin, e.g. ALB=Albania, AFG=Afghanistan, AZE=Azerbaijan, BIH=Bosnia and Herzegovina, CHN=China, DEU=Deutschland, DZA= Algeria, EGY= Egypt, ETH= Ethiopia, GEO=Georgia, GRC=Greece, IND= India, IRN= Iran, IRQ= Iraq, JOR= Jordan, LBN= Lebanon, LBY= Libya, MAR= Morocco, OMN= Oman, PAK=Pakistan, PAL= Palestine, RUS= Russia, SAU=Saudi Arabia, SYR= Syria, SCG=Serbia and montenego, TJK= Tajikistan, TKM=Turkmenistan, TUN= Tunis, TUR=Turkey, UZB= Uzbekistan, YEM=Yemen, UNK= Unknown.

Table S4 SSR marker information used in the evaluation of population structure

Chromosome	Marker name	Forward primer sequence (5'→3')	Revers primer sequence (5'→3')
1H	Bmac90	ACATCAACCCTCCTGCTC	CCGCACATAGTGGTTACATC
1H	Bmag105	AATCAGACCCATCAGAGGT	CCGGTCTCATAGAAATGG
1H	HVALAAT	TACATACAACCCTCATGGG	AAGGATGACATGGCTTTG
2H	Bmag125	AATTAGCGAGAACAAAATCAC	AGATAACGATGCACCACC
2H	Ebmac415	GAAACCCATCATAGCAGC	AAACAGCAGCAAGAGGAG
2H	HVBKASI	ATTGGCGTGACCGATATTTATGTTCA	CAAAACTGCAGCTAAGCAGGGGAACA
3H	Bmag13	AAGGGGAATCAAAATGGGAG	TCGAATAGGTCTCCGAAGAA
3H	HVLTPPB	AGACGCTGAGTACGTTGAG	CAAAGTACAACAAACTCACGA
3H	Hvm33	ATATTAAAAAAGGTGGAAAGCC	CACGCCCTCTCCCTAGAT
4H	Bmac30	CCCAATCGGAGTTACAGATG	GCCTCTCTGAGAATGGATC
4H	Gms89	TGAAGTGGAAGGCTTCGC	GCTCTCGTTGTGCGGAG
4H	HVMLOH1A	CCTCCCCTCTGATATGATAA	GTACAGACGGTTTAATTGTCC
5H	Bmag5	TCCATGATGATGTGTGCATAGA	CGGATCCCAACAAACACAC
5H	Gms1	CTGACCCTTTGCTTAACATGC	TCAGCGTGACAAACAATAAAGG
5H	HVLOX	CAGCATATCCATCTGATCTG	CACCCTTATTTATTGCCTTAA
6H	Bmac316	ATGGTAGAGGTCCCAACTG	ATCACTGCTGTGCCTAGC
6H	Hvm74	AGGAAGTCATTGCGTGAG	TGATCAAGAATGATAACATGG
6H	Scssr5599	TTCCATCATAACAGCAATGG	TTCGTCGAAGGCTATGTAGG
7H	Bmac156	AACCGAATGTATTCCTCTGTA	GCCAAACAACTATCGTGAC
7H	Bmag11	ACAAAAACACCGCAAAGAAGA	GCTAGTACCTAGATGACCCCC
7H	HVAMY2	CTGTAAGTGAGACAATCGACA	CAGTTGAACCCCTGAAAG

Primer sequences and locations for SSRs used in this study were obtained from the GrainGenes database (http://www.wheat.pw.usda.gov/ GG2/index.shtml).
